# Comparison of the Mg^2+^-Li^+^ Separation of Different Nanofiltration Membranes

**DOI:** 10.3390/membranes13090753

**Published:** 2023-08-24

**Authors:** Tingting Li, Yueyu Liu, Chandrasekar Srinivasakannan, Xiaobin Jiang, Ning Zhang, Guoli Zhou, Shaohua Yin, Shiwei Li, Libo Zhang

**Affiliations:** 1 Faculty of Metallurgical and Energy Engineering, Kunming University of Science and Technology, Kunming 650093, China; litting1221@163.com (T.L.); 20212202171@stu.kust.edu.cn (Y.L.); 2 Chemical Engineering Department, Khalifa University of Science and Technology, Abu Dhabi 999041, United Arab Emirates; csrinivasakannan@pi.ac.ae; 3 State Key Laboratory of Fine Chemicals, Frontier Science Center for Smart Materials, School of Chemical Engineering, Dalian University of Technology, Dalian 116023, China; xbjiang@dlut.edu.cn (X.J.); zhangning@dlut.edu.cn (N.Z.); 4 School of Chemical Engineering, Zhengzhou University, Zhengzhou 450001, China; zglcumt@126.com

**Keywords:** salt-lake brine, monovalent, multi-valent cations, ion interception rate

## Abstract

Nanofiltration application for the separation of Mg^2+^-Li^+^ from salt-lake brines was attempted in the present work. Four different nanofiltration membranes identified in the manuscript as DL, DK, NF-270, and NF-90 were used to treat salt brine with a magnesium to lithium ratio (MLR) of 61, additionally contaminated by the other ions such as Na^+^, K^+^, Ca^2+^, etc. The effect of the dilution factor, operating pressure, circulation rate, and feed pH were assessed to identify the optimal operating conditions for each membrane based on the retention efficiency of each ion. The results showed an insignificant effect of Ca^2+^ on the retention performance of Mg^2+^-Li^+^. Na^+^ and K^+^ had a smaller hydration radius and larger diffusion coefficient, which competed with Li^+^ and altered the separation of Mg^2+^-Li^+^. Under the optimal conditions (dilution factor: 40; operating pressure: 1.2 MPa; circulation flow rate: 500 L/h; pH: 7), the retention efficiency of lithium was as low as 5.17%, separation factor (SF) was as low as 0.074, and the MLR in the permeate reduced to 0.088.

## 1. Introduction

Lithium is a key raw material for energy storage batteries. The proportion of motor vehicles operated with rechargeable batteries is increasing year on year, which drives the demand for lithium and widespread attention on lithium metal extraction [1,2]. The prime source of lithium is ore, which continues to decline due to huge exploitation [3,4]. The exploitation of prime lithium ore resources such as spodumene and lepidolite has been widely documented [5]. Because the ore is a non-sustainable source and due to the drastic decline in the lithium resource reserves, the industry and scientific society efforts are persistent for alternative resources [6]. These persistent effects have led to the development of newer resources, of which salt lakes stand out in recent years [7]. Lithium resources in salt lakes account for nearly 70% of the basic reserves in 2021 [8,9]. China has the third largest lithium reserves in the world, of which 71% is accounted for in the salt lakes. Hence, the lithium resources from salt lakes are expected to be the main source in the near future catering to the increasing local and global demand [10].

The extraction of lithium from salt lakes is marred by two issues. Firstly, the Mg^2+^/Li^+^ ratio of Chinese salt lake brine (MLR = 1577) is too high compared to lithium-rich salt lakes elsewhere (MLR = 1.37) [11,12]. The efficient enrichment of lithium resources is hampered by the similar properties of magnesium and lithium [8,13]. Secondly, due to the presence of a large number of other ions (such as Na^+^, K^+^, Ca^2+^, Ba^2+^, etc.), the separation difficulty is added by the ionic valence as well as high concentration (0.0004-0.112 M). [14]. Therefore, it is necessary to understand the effects of other ions on host ions, so as to develop efficient separation processes.

At present, a variety of methods have been reported for extracting lithium from salt lakes, which include precipitation, adsorption, extraction, and membrane methods [15]. The precipitation method precipitates contaminant ions and enriches lithium by evaporation. It is a simple process being widely adopted in industry, but applicable only for brine with low MLR, and the high cost of the precipitation renders it unfeasible [16,17]. In comparison, the adsorption can handle high MLR brine, but is limited by the low adsorption capacity of the adsorbents and the need of adopting the cyclic adsorption–desorption process [18]. The extraction process can separate lithium using an appropriate solvent, but limited by the difficulties of phase separation and the solvent losses [19]. As an emerging alternative, nanofiltration is a pressure-driven membrane separation process, which offers the advantages of high separation efficiency, being less energy-intensive, being environmentally benign, and easy operation at large scales [20,21]. Based on the Don-nan repulsion and pore screening effect, a high interception efficiency for polyvalent ions and good permeability can be achieved for the effective separation of Mg^2+^-Li^+^ [22,23]. Thus, nanofiltration has the potential to serve to be a better separation process to affect the lithium extraction from salt lakes.

Recently, commercial polyamide nanofiltration membranes are widely use for the separation of Mg^2+^-Li^+^ from salt lakes, commonly known as NF, DK, and DL [24]. Ji et al. [25] had confirmed that high MLR is not conducive for separation using the DK-2540 membrane, as a significant competition of Na^+^, K^+^, and Li^+^ while Ca^2+^ is not significant. Sun et al. [26] had compared the separation performance of DL-2540 and DK-2540 membranes and reported similar performance of both membranes. The results showed high separation factor at low MLR, and the separation factor is insignificant while the MLR is higher than 20. Pramanik et al. [27] had compared the Mg^2+^-Li^+^ separation performance of NF-90 and NF-270 and reported that NF-90 reduces MLR from 10 to 0.19 (Li^+^ extraction 23%), while NF-270 from 10 to 2.1 (Li^+^ extraction 44%).

The present work attempted to assess the Mg^2+^-Li^+^ separation performance of four nanofiltration membranes, namely DL, DK, NF-270, and NF-90, due to the understanding of the hydration radius of each ion and the basic properties of the NF membrane (MWCO, MgSO_4_ retention rate, etc.). The effects of different operating parameters, namely, the dilution, operating pressure (transmembrane pressure), circulation flow rate, and pH, were systematically investigated on the four objective functions, namely, ion retention efficiency, membrane flux, SF, and MLR. The effect of impurity (Na^+^, K^+^, Ca^2+^, etc.) on the retention efficiency of Mg^2+^-Li^+^ was also analyzed by the Donnan repulsion effect, pore size sieving effect, and dilution effect.

## 2. Experimental

### 2.1. Experimental Materials and Device

The ion composition of the simulated brine resembled the brine of Yiliping Salt Lake, which had a MLR of 61, and the specific ion composition was shown in Table 1. The reagents used in the preparation of the simulated brine were all analytical grade (purity ≥ 99.98%), including lithium chloride (LiCl·H_2_O), magnesium chloride (MgCl_2_), magnesium sulfate (MgSO_4_), potassium chloride (KCl), sodium chloride (NaCl) and calcium chloride (CaCl_2_). All the aforementioned chemicals were obtained from Sinopharm Chemical Reagent Co., Ltd., (Shanghai, China).

**Figure 1:**
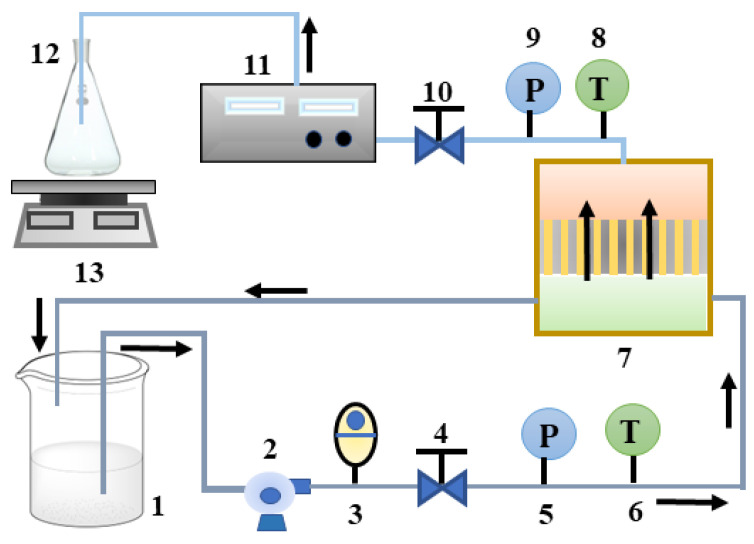
Experimental flow chart. (1. raw material drum; 2. pressure pump; 3. control valve; 4, 10. pressure valve; 5, 9. pressure gauge 6, 8. thermometer 7. membrane block 11. conductivity meter 12. permeate collection bottle 13. electronic scale).

The triple high-pressure flat membrane test equipment was used for nanofiltration separation experiments. The nanofiltration separation process schematic was shown in Figure 1. The feed solution was pumped to the module by a high-pressure pump, where a small part of them passed through the membrane to the permeate side and most of them returned to the feedstock barrel (retentate). Once the conductivity of the permeate was stable, it would be collected and the membrane flux would be calculated. During the experiment, the temperature of the feedstock barrel was kept constant with a cooling arrangement. The inlet and outlet pressure were controlled by the pressure regulating valve while the feed flow rate was through the control valve.

### 2.2. Experimental Membrane

The commercial nanofiltration membranes used in the following experiments were supplied by Dow, and their basic properties and operating range were shown in Table 2 [28].

Four nanofiltration membranes (DL, DK, NF 270, NF 90) with Mg^2+^-Li^+^ separation properties were initially screened through the understanding of the hydration radius of each ion and the basic properties of the NF membrane (molecular weight cutoff (MWCO), MgSO_4_ retention rate, etc.). The effects of different operating parameters, namely, the dilution ratio, solution pH, operating pressure, and circulating flow on the separation of Mg^2+^-Li^+^, were assessed for all four membranes chosen in the study. The experiments were conducted within the operating range of commercial nanofiltration membranes (temperature = 25 ± 0.1 °C; pressure = 0–2.5 MPa; feed liquid pH = 3–11).

### 2.3. Analysis and Characterization Methods

Inductively Coupled Plasma Emission Spectrometer (ICP-OES, 7500CS, Agilent Technologies, Wilmington, DE, USA) was used to measure ion concentrations (such as Li^+^, Na^+^, K^+^, Mg^2+^, Ca^2+^) [27]. The pH meter (PHS-3E, Thunderstorm, Shanghai, China) was used to measure the pH of the raw brine. The field emission Scanning Electron Microscope (SEM, Hitachi SU8010, Japan) was used to characterize the surface and side morphology of the nanofiltration membrane. The surface roughness of the nanofiltration membrane was obtained by Atomic Force Microscopy (AFM, Shimadzu SPM-9500 J3, Kyoto, Japan). The contact angle of the membranes was obtained by a contact angle measuring instrument (LSA60, LAUDA Scientific GmbH, Germany, Europe). The solid surface Zeta potential analyzer (SurPASS, Anton Paar, Austria) was used to study the charge properties of the membrane surface.)

### 2.4. Data Analysis

#### 2.4.1. Membrane Permeate Flux

Membrane flux is the volume of permeate passing through a unit membrane area per unit time [2], as shown in Equation (1):

(1) $$J_{v}=\frac{V}{At}$$

where: $J_{v}$ is the membrane flux (L·m^−2^·h^−1^); *V* is the permeate volume (L); *A* is the membrane area (m^2^); and *t* is the time required to collect permeate per unit volume (h).

The permeation volume flux refers to the volume of dialysate passing through the unit effective membrane area per unit time at a given pressure [29], such as Equation (2):

(2) $$J_{v}=L_{P}\left(\Delta P-\Delta \pi \right)$$

where $L_{P}$ is the permeability coefficient of the NF membrane (only related to temperature and membrane structure parameters); ΔP is the pressure difference on both sides of the membrane (bar); and Δπ is the osmotic pressure (bar). Studies have shown that the greater permeation flux, the better the membrane permeability. And Δπ = 0 when the solution to be tested is pure water according to the Dissolution-Diffusion Model, pure water permeation flux is show in Equation (3).

(3) $$J_{v}=L_{P}\cdot \Delta P$$

**Figure 2:**
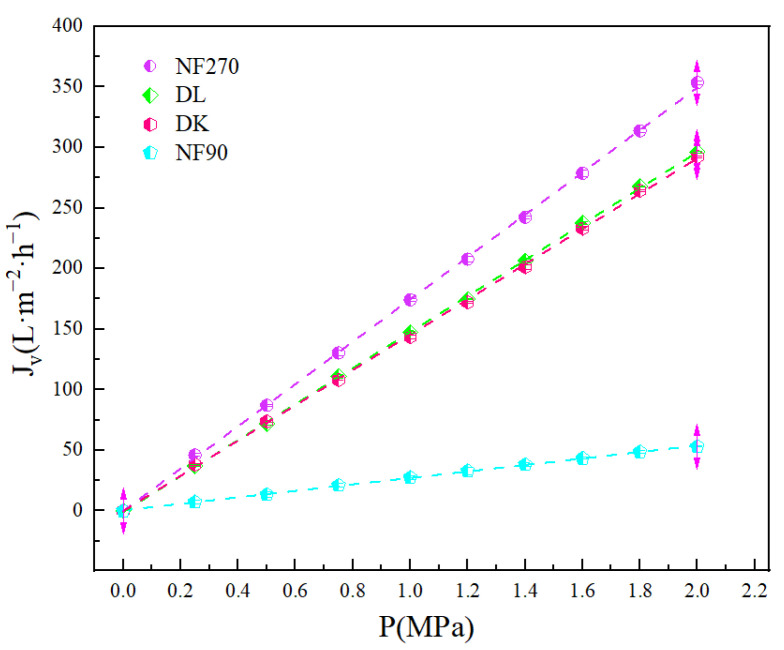
Response of membrane pure water flux with operating pressure.

The relationship between pure water permeation flux and operating pressure of various nanofiltration membranes was obtained. Some test sites deviated due to the instability of the nanofiltration membrane and the nanofiltration equipment under high pressure conditions while the membrane maintained good stability in the low-pressure range, but the error was within the acceptable range (<5%), which had little impact on the experimental results, as shown in Figure 2. The corresponding permeability coefficient of the membrane ($L_{P}$) was estimated by fitting the data and the performance was in the increasing order of NF270 ($L_{P}$: 174.76) > DK (148.56) > DL (145.63) > NF90 (26.73). $L_{P}$ refers to the influence of pressure on membrane flux; the larger the $L_{P}$ is, the more significant effect of pressure on membrane flux is.

#### 2.4.2. Salt Retention

Salt rejection efficiency ($R_{S}$) refers to the ratio of the concentration of the solute in the permeate to the concentration of the solute in the feed, as shown in Equation (4) [30].

(4) $$R_{S}=\left(1-\frac{C_{p}}{C_{f}}\right)\times 100\%$$

where: $C_{p}$ and $C_{f}$ are the permeate and feed concentrations, respectively (unit: g/L). Studies have shown that the higher the salt rejection, the better the separation performance of the nanofiltration membrane.

#### 2.4.3. Separation Factor of Magnesium and Lithium (SF)

The separation factor refers to the ratio of permeate to feed liquid MLR, as shown in Equation (5).

(5) $$SF_{Mg/Li}=\frac{\left(C_{Mg,p}/C_{Li,p}\right)}{\left(C_{Mg,f}/C_{Li,f}\right)}$$

where $C_{Mg,p}$ and $C_{Li,p}$ are the concentrations of Mg^2+^ and Li^+^ (g/L) in the permeate. $C_{Mg,f}$ and $C_{Li,f}$ are the concentrations of Mg^2+^ and Li^+^ (g/L) in raw brine. The separation factor of 1 indicates no separation, while lower than 1 shows preferential permeation of Li^+^ and the lower the separation factor is, the better the separation performance is.

## 3. Results and Discussion

### 3.1. Effect of Dilution Factor on the Separation Performance of Mg^2+^-Li^+^

**Figure 3:**
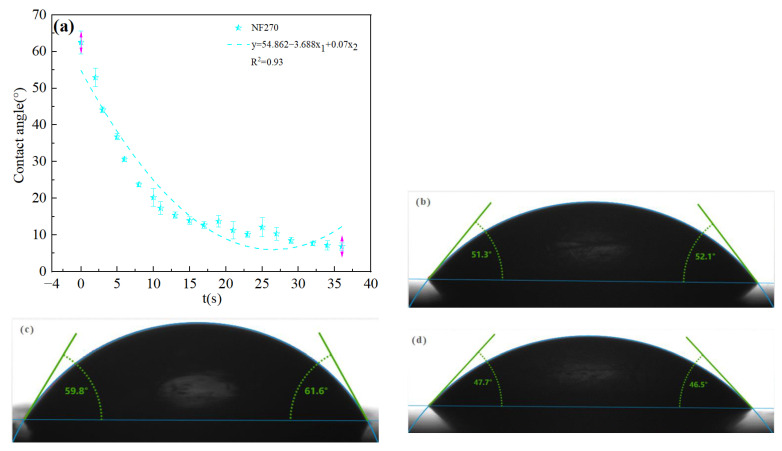
(**a**) Dynamic contact angle of DL film and contact angle diagram of (**b**) DK, (**c**) NF270, (**d**) NF90.

The high salt contents of salt lake brine facilitates the easy formation of ion pairs, ion groups, and molecules contributing to the steric hindrance effect [31]. Ions attach to the membrane surface and block the membrane pores during the separation process, thereby reducing membrane flux and increasing membrane fouling [32]. Therefore, the directly use of nanofiltration technology is not conducive to separation, so it is necessary to dilute the raw material brine so as to reduce the membrane pollution. The hydrophilicity and hydrophobicity of the membrane are important parameters that affect the membrane flux and antifouling performance [33]. Due to the good water absorption of the DL film and the rapid disappearance of droplets, the contact angle could not be accurately measured during the detection process, so the dynamic contact angle fitting curve was used to observe the contact angle. The dynamic contact angle reflects the wetting process of a droplet contacting a membrane surface, including diffusion and osmosis. The contact angles of four commercial nanofiltration membranes were estimated and presented in Figure 3: DL (62.5°) > NF270 (60.7°) > DK (51.7°) > NF90 (47.1°). A contact angle of less than 90° indicates the membrane to be hydrophilic, having better surface wettability, reducing surface adhesion and fouling tendency contributing to increases ions permeability [34]. As the dilution of the feed solution was expected to reduce the adhesion of ions on the membrane surface, the effect of dilution ratios covering a range of 25 to 50 on the separation performance of Mg^2+^-Li^+^ was assessed with the indoor temperature of 25 ± 0.1 °C. Each experimental data point had been validated by at least three sets of parallel experiments.

**Figure 4:**
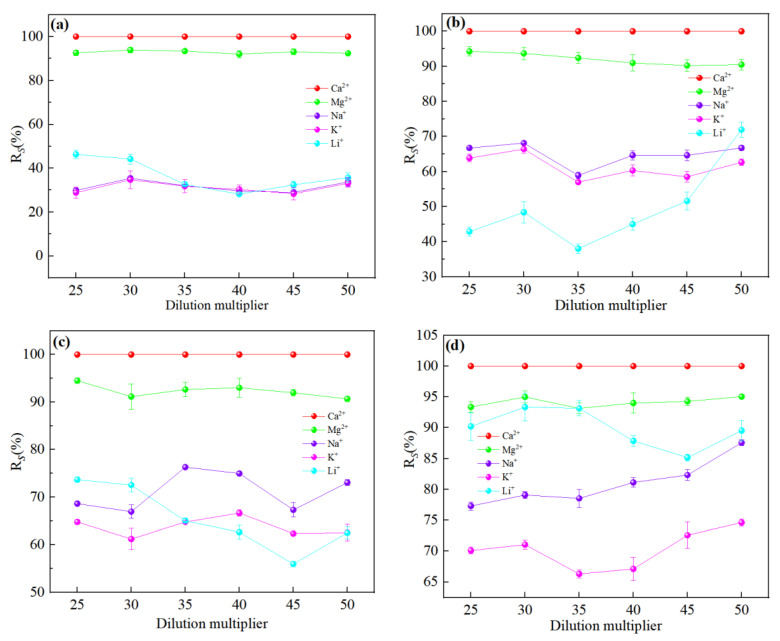
Retention efficiency (%) of each ion at different dilution multiplier: (**a**) DL; (**b**) DK; (**c**) NF270; (**d**) NF90. The experimental conditions of the four types of nanofiltration membranes were: operating pressure = 0.5 MPa, circulation flow rate = 600 L/h, and feed pH = 7.

It could be seen from Figure 4 that the order of interception effect of nanofiltration membranes on ions was: R(Ca^2+^) > R(Mg^2+^) > R(Na^+^) ≈ R(K^+^) > R(Li^+^), except for the NF90 membrane. It was well known from the Donnan repulsion effect that negatively charged membranes were more attractive to divalent cations (Mg^2+^, Ca^2+^) than monovalent cations (Na^+^, K^+^, Li^+^), and facilitated the preferential movement of divalent cations to the membrane surface. It could be seen from Table 3 that divalent cations could not pass through the membrane pores smoothly due to the large hydration radius, while monovalent cations could pass through the membrane pores smoothly. The interception of metal cations was related to the degree of enrichment on the membrane surface and was proportional to the ion concentration. Since the concentration of Ca^2+^ was much lower than that of Mg^2+^, the competitive permeability of Ca^2+^ was nearly zero. Comparatively, as the Mg^2+^ concentration was high, its permeability was around 10% as a small part of ions still could enter the membrane pores through ‘dehydration’. Since, Na^+^ and K^+^ had smaller hydration radii and larger diffusion coefficients compared to Li^+^, their permeation flux was higher than Li^+^ so that Li^+^ transmission was limited. However, due to the large concentration of Na^+^ and K^+^, there were still a large number of ions that did not pass through, so the interception rate was still above Li^+^. But in the Figure 4d, we could find that because the pore radius was closed to the hydration radius of Li^+^ for NF 90 membrane, a significant aperture screening effect was led, and the transmittance of Li^+^ was limited. Although there was still some Li^+^ that could penetrate through the membrane pores by removing of the hydrated layer, a large amount of Li^+^ were still retained in the raw material liquid, which made the interception rate of NF 90 membrane to Li^+^ higher than that of Na^+^ and K^+^, which was not conducive to separation.

**Figure 5:**
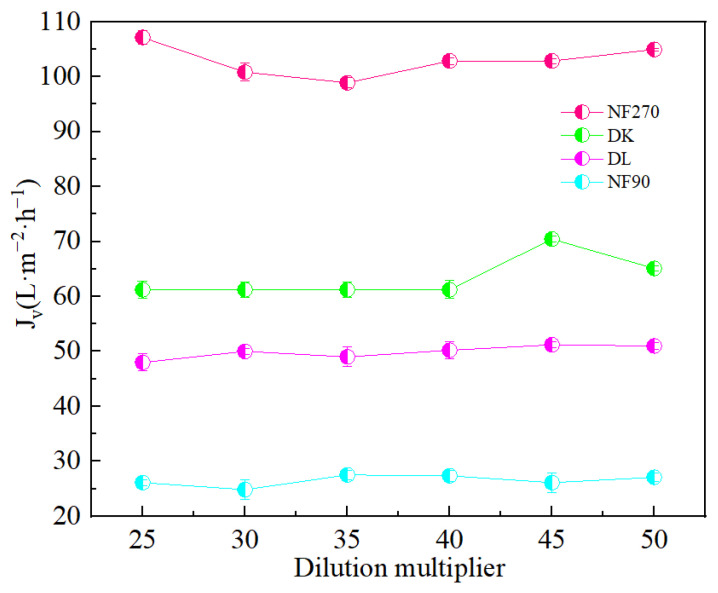
Effect of different dilution multipliers on the membrane flux (*J_v_*).

**Figure 6:**
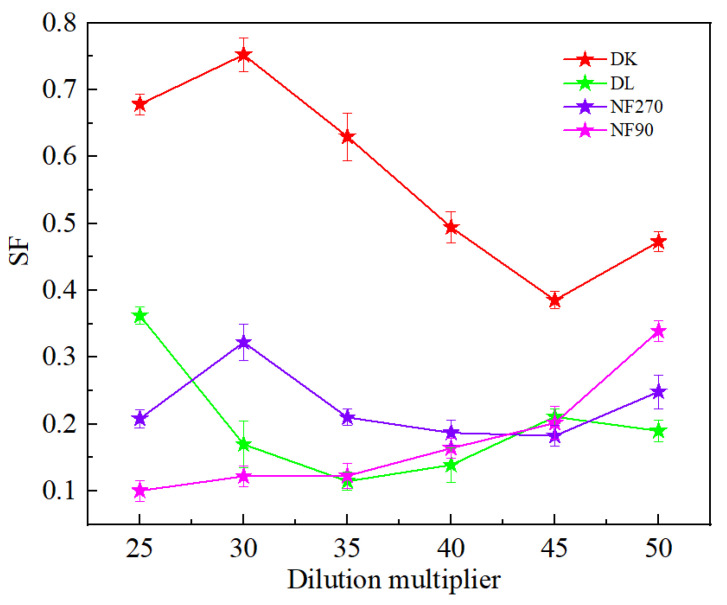
Effect of different dilution multipliers on the SF.

Figure 5 showed that the dilution factor had little effect on the permeate flux of the membrane. Although the NF90 membrane had the smallest contact angle and the strongest hydrophilicity, due to the smaller membrane pore size, the solute flux was lower than the water flux, and resulted in the largest. However, the separation factor showed a significant reduction in the increase in the dilution factor, which was conducive to separation. The separation factors of all membranes were different, but less than 1, indicated its effectiveness on the separation of Mg^2+^-Li^+^ according to Figure 6.

### 3.2. Effect of Operating Pressure on the Separation Performance of Mg^2+^-Li^+^

The operating pressure is known to strongly influence the membrane flux as it is the driving force for separation and energy consumption. The increase in the transmembrane pressure increases both solvent and solute fluxes. However, the solvent flux increases much more than the solute flux resulting in a more diluted permeate, and a higher solute rejection. Experiments were carried out at low pressure (0–2.5 MPa) to attenuate the effect of the dilution effect as well as the lower power consumption. Each experimental data point had been validated by at least three sets of parallel experiments.

**Figure 7:**
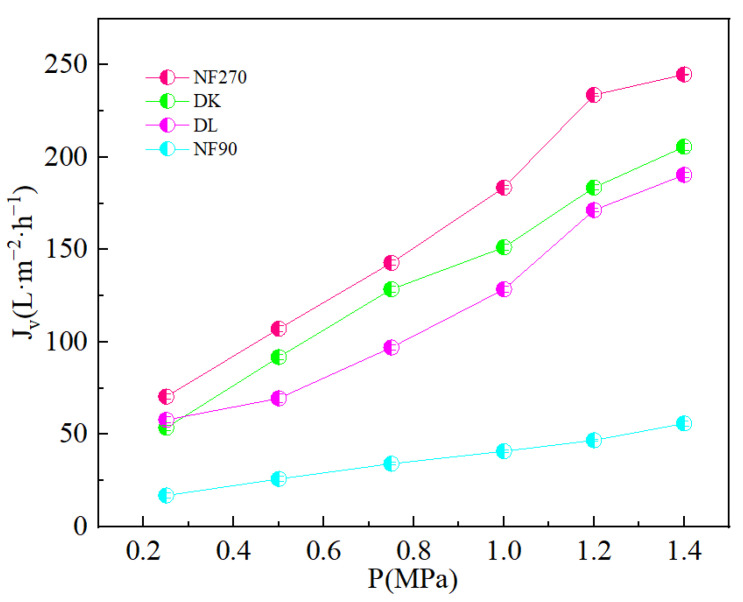
Effect of different transmembrane pressure on the membrane flux (*J_v_*).

The membrane flux is closely related to the total separation resistance, which is the function of the parameters as shown in Equations (6) and (7), according to Darcy’s law.

(6) $$J_{v}=\frac{\Delta P\;-\;\Delta \pi }{{\mu R}_{t}}$$

(7) $$R_{t}=R_{m}+R_{cp}+R_{ad}+R_{f}$$

where: *µ* is the dynamic viscosity of the raw material solution (Pa · s); *R_t_* is the total separation resistance (m^−1^); $R_{m}$ is the membrane surface resistance; $R_{cp}$ is the concentration polarization resistance; $R_{ad}$ is the adsorption resistance; and $R_{f}$ is the fouling resistance. With the increase in pressure, the kinetic force of ions across the membrane increases, which weakens the Donnan repulsion effect. At the same time, due to the cross-flow filtration method, the residence time of ions on the membrane surface is shortened, which weakens the concentration polarization effect. The reduction in the enrichment of ions on the membrane surface reduces the fouling resistance and the resistance of ions adsorbed on the membrane surface, all of which contribute to the reduction in separation resistance and increase in permeate flux. Figure 7 showed an increase in the permeate flux with an increase in operating pressure and an increase in the order: NF270 > DK > DL > NF90.

**Figure 8:**
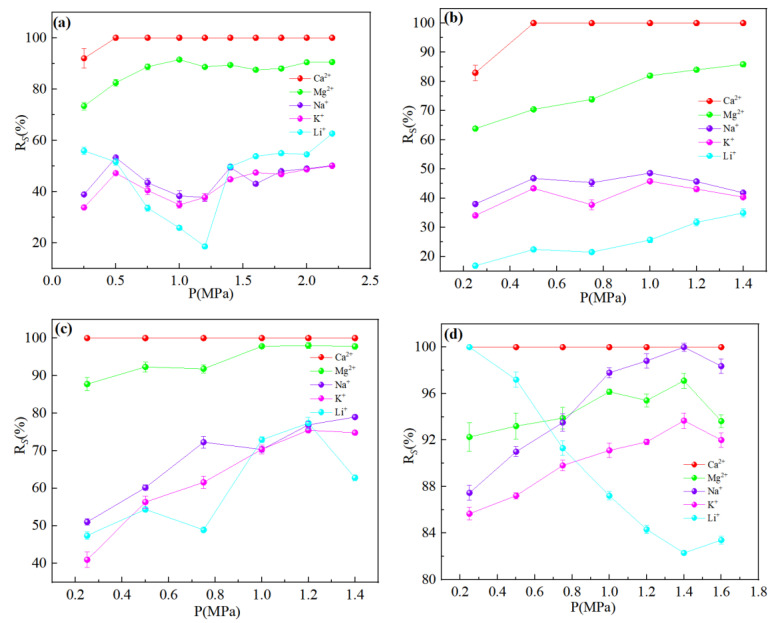
Retention efficiency (%) of each ion at different transmembrane pressure: (**a**) DL; (**b**) DK; (**c**) NF270; (**d**) NF90. The experimental conditions: DL (dilution multiplier = 40, circulation flow rate = 600 L/h, and feed pH = 7); DK (dilution multiplier = 35, circulation flow rate = 600 L/h, and feed pH = 7); NF270 (dilution multiplier = 40, circulation flow rate = 600 L/h, and feed pH = 7); NF90 (dilution multiplier = 45, circulation flow rate = 600 L/h, and feed pH = 7).

**Figure 9:**
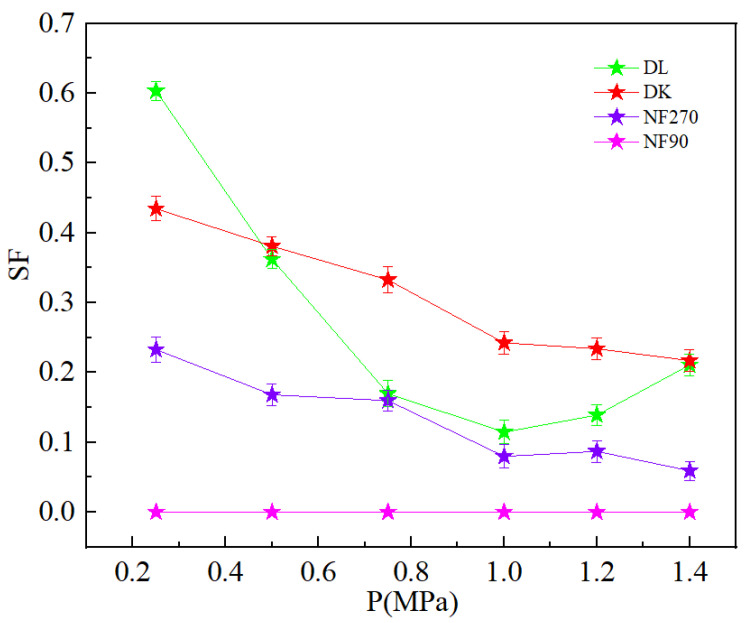
Effect of different pressure on the SF.

Figure 8 showed the relationship between the percentage of ion rejection and operating pressure. The ions in the dialysate were concentrated, which is because the water flux and membrane flux of each ion were not very different under the low pressure, which resulted in a low interception rate of each ion. The driving force of water molecules and the ions through the membrane increased with the increasing pressure, but because the increase in water flux was significantly higher than the flux of each ion, the concentration of each ion in the dialysate was diluted and the interception rate increased [35]. The dilution effect was unfavorable for ion separation, so the separation process was not good to use large pressure. The Li^+^ interception rate decreased with the increase in pressure as shown in Figure 8d. Li^+^ was unable to obtain enough power to pass through the membrane hole under the low pressure, which led to a high interception rate. With the increasing pressure, Li^+^ obtained sufficient energy to remove the hydration layer and through the membrane, which led to a decrease in the interception rate. Figure 9 showed the SF decreased with an increase in the operating pressure, but the larger pressure could cause significant dilution effect, which was not conducive to ion interception. Therefore, the best operating pressure should be selected according to the interception performance of different nanofiltration membranes.

### 3.3. Effect of Circulating Flow on the Separation Performance of Mg^2+^-Li^+^

The circulating flow rate of the feed liquid is known to affect the concentration polarization on the membrane surface, thereby it will affect the separation of Mg^2+^-Li^+^. In order to weakened the concentration polarization effect due to high ion concentration, the circulation flow rate of the nanofiltration process was maintained lower than 600 L/h. Each experimental data point had been validated by at least three sets of parallel experiments.

**Figure 10:**
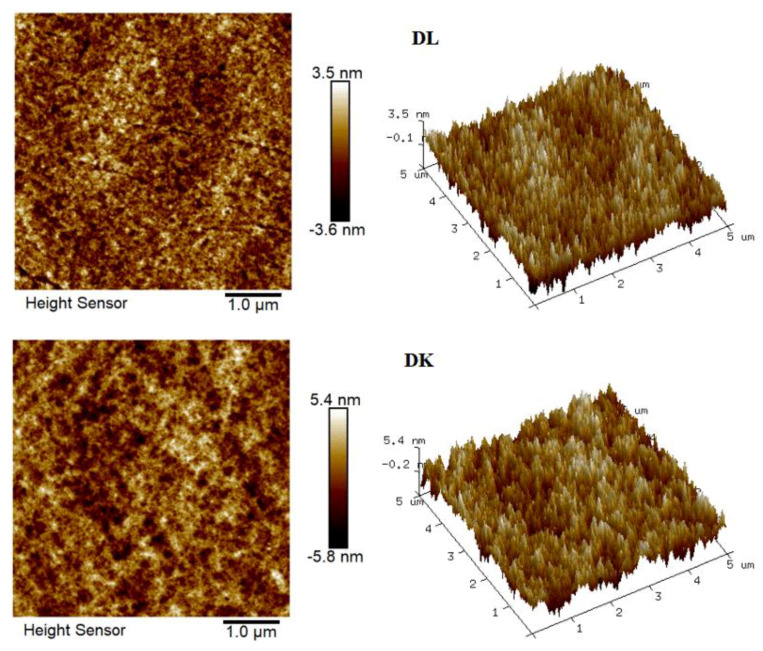

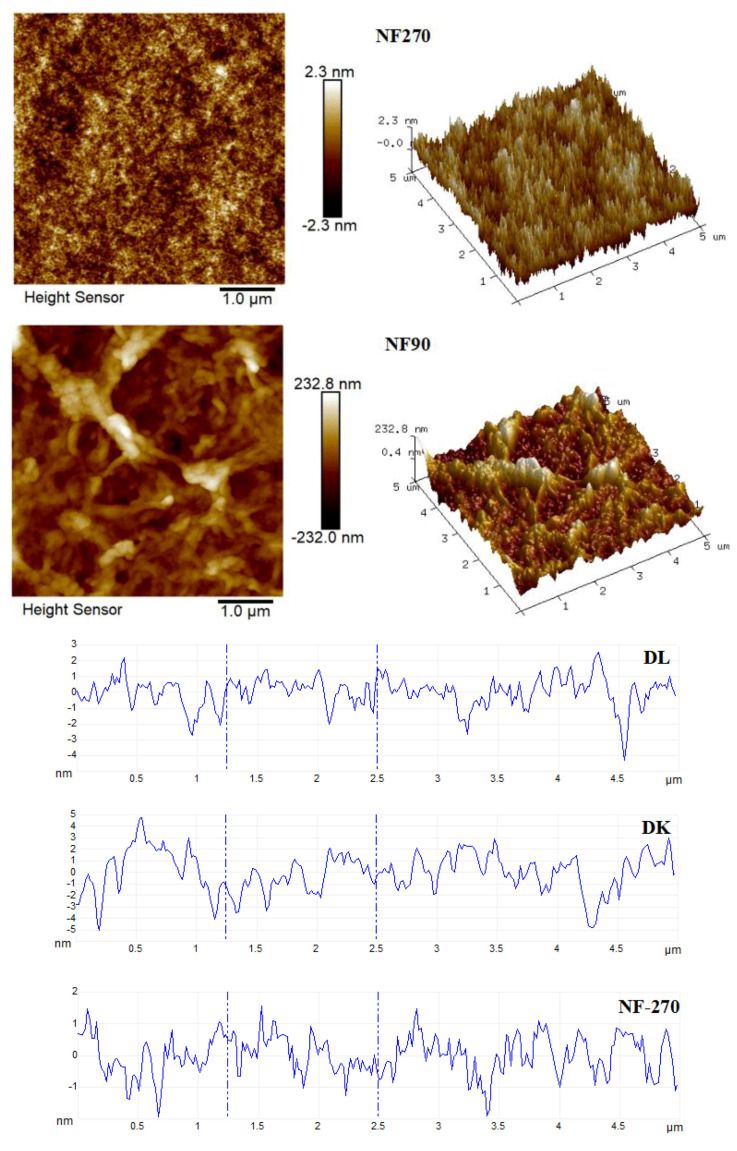

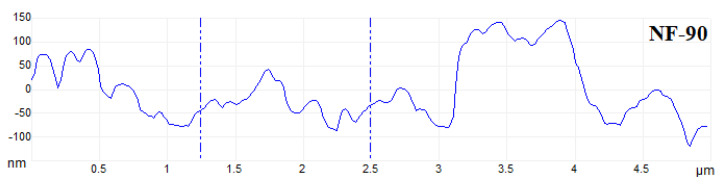
AFM of membranes.

**Figure 11:**
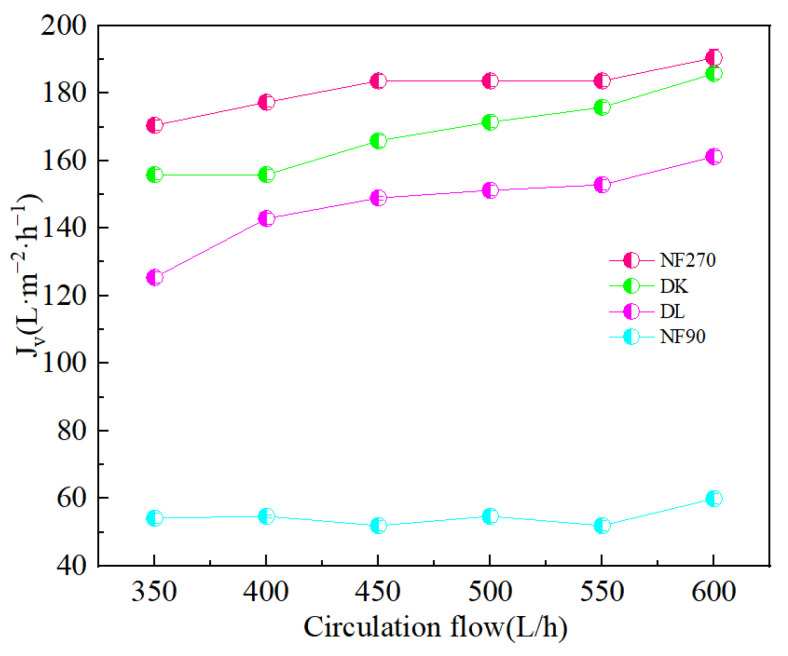
Effect of different circulation flow on the Membrane flux (*J_v_*).

The flow state of the feed liquid on the surface of the membrane was related to the roughness of the membrane surface [36]. Figure 10 showed the AFM images of the membranes, evidenced the variation in the surface roughness. The surface roughness was found to decrease in the order: NF90 (Ra = 42.2 nm) > DK (Ra = 1.31 nm) > DL (Ra = 1.0 nm) > NF270 (Ra = 0.52 nm). The membrane surface had a typical ‘ridge-valley’ structure, and the peak density was found in descending order of NF270 > DL > DK > NF90. The ‘nanoscale’ structure on the surface could accelerate the microscopic mixing of the feed liquid. The hydrodynamic condition was improved by the enhance of the interface contact area, and the permeate flux was increased as a result, as shown in Figure 11.

**Figure 12:**
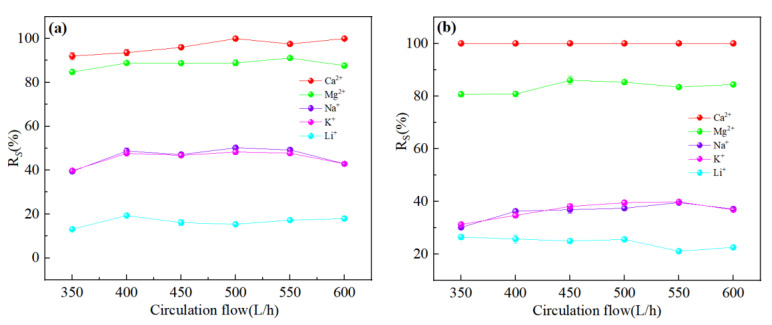

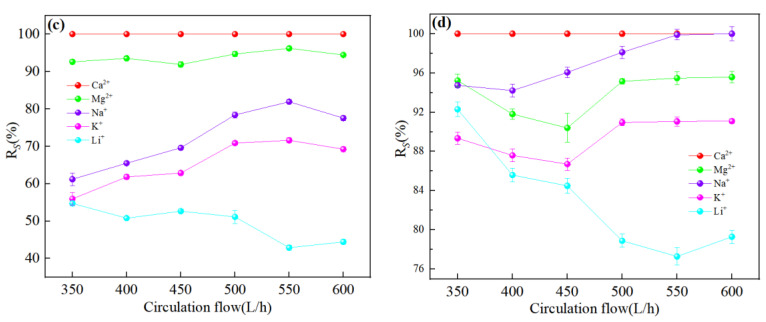
Retention efficiency (%) of each ion at different circulation flow: (**a**) DL; (**b**) DK; (**c**) NF270; (**d**) NF90. The experimental conditions: DL (dilution multiplier = 40, operating pressure = 1.2 MPa, and feed pH = 7); DK (dilution multiplier = 35, operating pressure = 1.0 MPa, and feed pH = 7); NF270 (dilution multiplier = 40, operating pressure = 0.75 MPa, and feed pH = 7); NF90 (dilution multiplier = 45, operating pressure = 1.4 MPa, and feed pH = 7).

**Figure 13:**
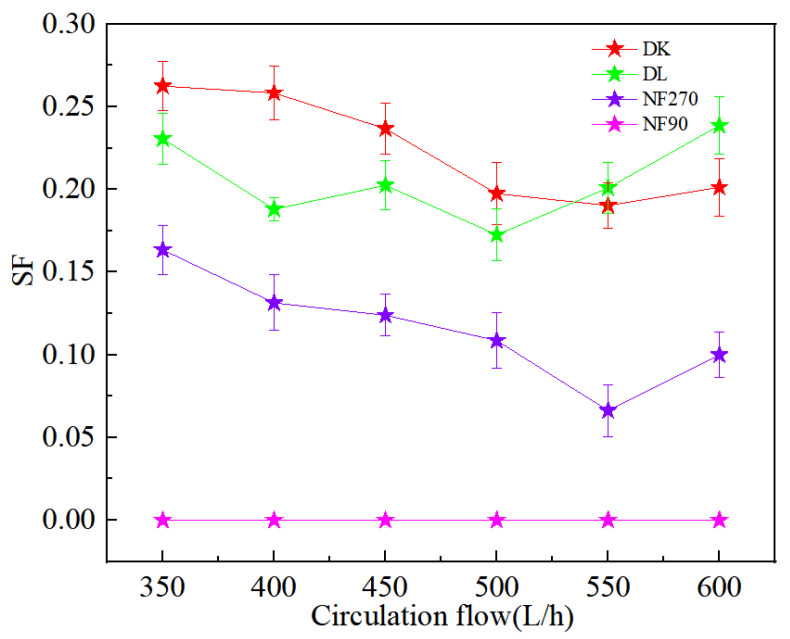
Effect of different circulation flow on the SF.

Figure 12a,b showed the ion rejection efficiency of DL and DK membranes were stable with an increase in circulation rate. This could be attributed to the decrease in the aggregation of ions on the membrane surface, contributed to a decrease in membrane fouling and concentration polarization effects. The reduction of membrane fouling would be contributed to an increase in ion flux, while weakening of the concentration polarization effect would reduce the driving force of ions, as a result of contradicted effects the rejection efficiency remains stable. It could be seen from Figure 12c,d that the Li^+^ rejection efficiency was opposite to that of Na^+^ and K^+^, which could be attributed to the reduction in the residence time of ions on the membrane surface, contributed to a reduction in the ion concentration, thereby reduced the competitive permeability of Na^+^ and K^+^. The rejection efficiency decreased as Li^+^ was more easily permeable through the membrane. The decrease in SF indicated better Mg^2+^-Li^+^ separation, as shown in Figure 13.

### 3.4. Effect of Brine pH on the Separation Performance of Mg^2+^-Li^+^

The pH of the feed solution will affect the membrane surface charge, thereby affecting the Donnan repulsion and the dielectric repulsion effect [37]. The pH of the feed solution was altered using NaOH and HCl (1 mol/L) and varied in the range from 3 to 11. Each experimental data point had been validated by at least three sets of parallel experiments.

**Figure 14:**
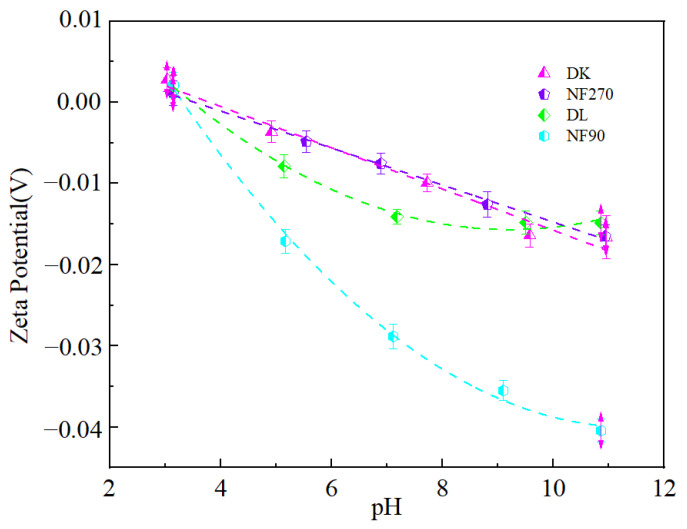
Zeta potential-pH relation chart.

The isoelectric point of the membrane (Zeta potential = 0) was shown in Figure 14. The decreasing zeta potential was of the order: DK (3.8) > NF270 (3.53) > DL (3.48) > NF90 (3.31). When the pH was lower than the isoelectric point, the surface of the membrane was positively charged, while it was negatively charged at a pH higher than the isoelectric point [38]. The characteristics of membrane surface charge were related to the dissociation of membrane surface groups and the adsorption of anions and cations (H^+^, OH^−^) in solution [39]. Since the nanofiltration membrane had a polyamide functional layer, the residual carboxyl functional group on the surface of the membrane dissociated when in contact with an aqueous solution and became negatively charged. However, the C_H+_ in the feed solution was relatively high at pH 3, and the accumulation of H^+^ would cause charge reversal. The zeta potential of the membrane surface decreased with the increase in pH. This was because the composition of salt-lake brine was complex, and there were a large number of anions (Cl^−^, SO_4_^2−^) in addition to metal cations. The dehydration ability of anions was stronger than that of cations, so they were more likely to lose binding. When the water reached the surface of the membrane, a large number of anions were adsorbed on the surface of the membrane, thereby increased the negative charge on the surface of the membrane.

**Figure 15:**
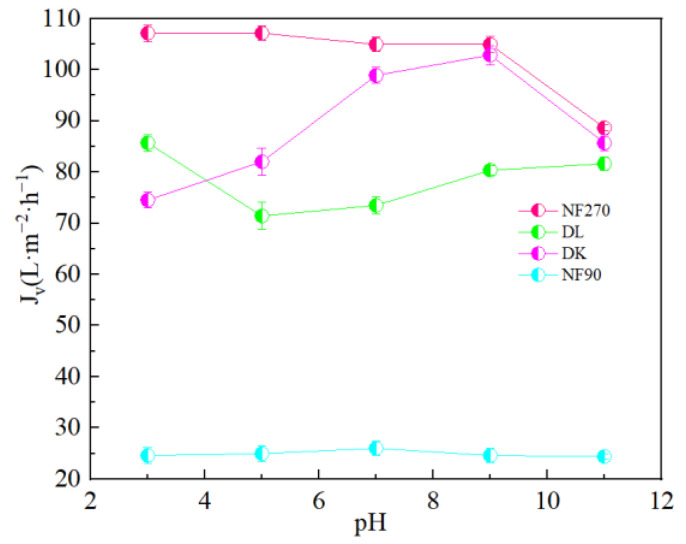
Effect of different pH on the membrane flux (*J_v_*).

**Figure 16:**
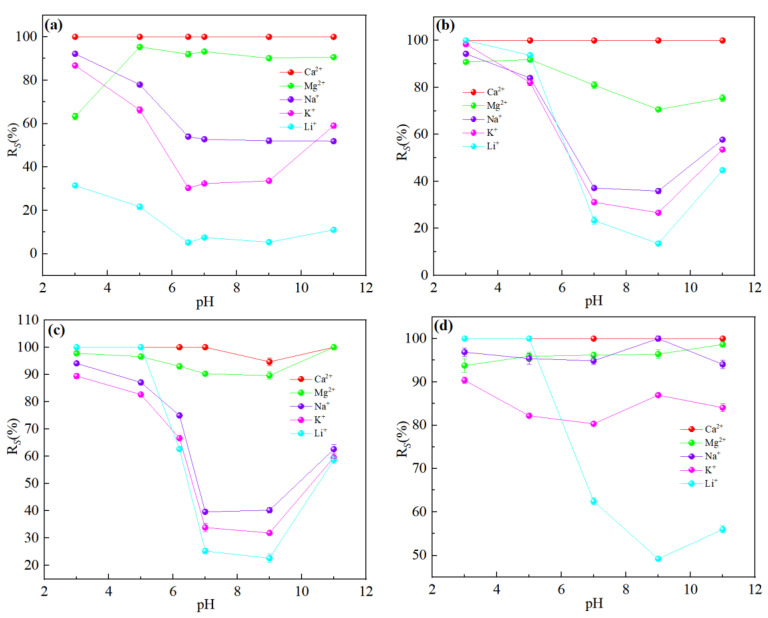
Retention efficiency (%) of each ion at different pH: (**a**) DL; (**b**) DK; (**c**) NF270; (**d**) NF90. The experimental conditions: DL (dilution multiplier = 40, operating pressure = 1.2 MPa, and circulation flow rate = 500 L/h); DK (dilution multiplier = 35, operating pressure = 1.0 MPa, and circulation flow rate = 550 L/h); NF270 (dilution multiplier = 40, operating pressure = 0.75 MPa, and circulation flow rate = 550 L/h); NF90 (dilution multiplier = 45, operating pressure = 1.4 MPa, and circulation flow rate = 600 L/h).

**Figure 17:**
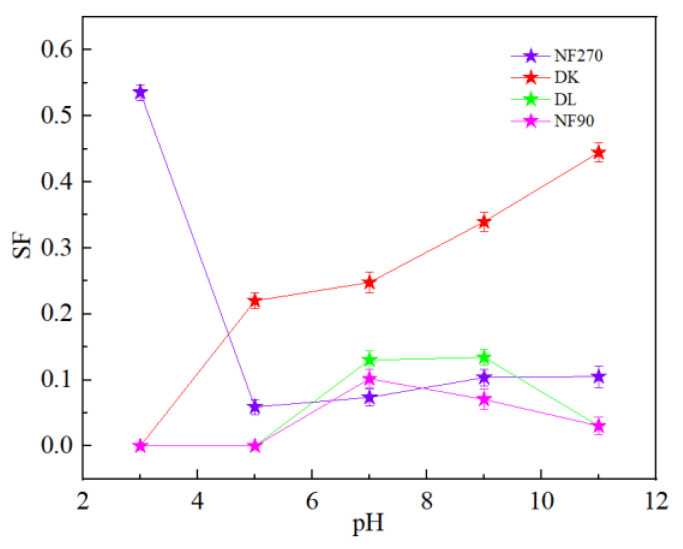
Effect of different pH on the SF.

It could be seen from Figure 15 that pH had little effect on the flux of NF270 and NF90 membranes, while DL and DK membranes showed different trends. As shown in Figure 16, divalent cations were less affected by the pH, and monovalent cations showed a ‘concave’ shape change trend with the pH. When the pH was lower than the isoelectric point, the solution contained excess H^+^. Since H^+^ has a smaller hydration radius and a larger diffusion coefficient, it was easier to permeate the membrane and limited the penetration of other monovalent cations, resulted in a higher rejection. When the pH of the feed solution was higher than the isoelectric point, as the pH increased, the C_H+_ in the feed solution gradually decreased while the C_OH_- gradually increased, which led to the negative charge density on the surface of the membrane increasing and needing to attract cations to maintain electrical neutrality. Based on the Donnan effect, the stronger the negative charge on the membrane surface, the greater the attractive effect on the multivalent cations than on the monovalent cations is, which was not conducive to separation, so the pH of the feed solution could be kept at 7–9 to obtain smaller SF, as shown in Figure 17.

To sum up, due to the influence of the hydration radius of ions and the interaction between ions caused by the complex composition of brine, the separation process would be interfered by the impurity ions. This resulted a significant increase at the rejection of Li^+^, so the separation effect was slightly different from that of the two-component solution of Mg^2+^-Li^+^ [28]. The best operating conditions of each membrane were obtained through the above experiments as shown in Table 4. The SFs of the four commercial nanofiltration membranes were all less than 1, so the nanofiltration process achieved the enrichment of Li^+^, and the smaller the SF and dialysate MLR, the better the separation of Mg^2+^-Li^+^. Therefore, the DL membrane was more suitable for the separation of Mg^2+^-Li^+^ in salt-lake brine.

## 4. Conclusions

Nanofiltration was successfully applied for the separation of Mg^2+^-Li^+^ and four commercial nanofiltration membranes were utilized to separate salt-lake brine containing various ions such as Li^+^, Na^+^, K^+^, Mg^2+^, Ca^2+^, etc. The results indicated that, among the four different nanofiltration membranes, the performance of the DL membrane was the best one under the optimal operating conditions of a dilution factor of 40; operating pressure of 1.2 MPa; circulation flow rate of 500 L/h, and feed pH of 7. The SF was as low as 0.074, and the MLR (a ratio of molar concentrations) was reduced from 17.406 to 0.088, offering the best separation of Mg^2+^-Li^+^, which laid a foundation for further scale-up and commercial adoption of the process.

The salt contents of salt-lake brine are complex and the concentration is large, so the direct use of nanofiltration membrane separation technology is easy to cause membrane pollution, thus the separation efficiency is weakened and the cost increased. Therefore, the optimal concentration of each impurity ion in the salt-lake brine can be studied, and the pretreatment technology can be adopted to remove some impurity ions, so as to reduce the membrane pollution problem of nanofiltration technology and increase the separation effect.

## Figures and Tables

**Table 1:** Comparison of ion concentrations of the Yiliping Salt Lake with the simulated solution/wt%.

Ion Concentrations	Li^+^	Mg^2+^	K^+^	Na^+^	Ca^2+^	Cl^−^	SO_4_^2−^	MLR
Yiliping Salt Lake	0.021	1.28	0.91	2.58	0.016	14.97	2.88	60.95
simulated solution	0.021	1.28	0.91	2.58	0.016	6.54	2.88	60.95

**Table 2:** Application scope of commercial nanofiltration membrane with a negative charge.

Model	MWCO (Da)	Temperature (°C)	Pressure (MPa)	pH	Rejection of MgSO_4_
DL	924.21	90	3.5	1–11	≥98%
DK	947.15	10–50	3.5	2–11	≥94%
NF-270	865.80	45	3.5	3–10	≥97%
NF-90	1089.21	35	4.1	3–9	≥97%

**Table 3:** Ionic properties.

Ion Property	Li^+^	Mg^2+^	K^+^	Na^+^	Ca^2+^
ionic radius (r/nm)	0.094	0.072	0.149	0.117	0.099
Hydration radius (r_H_/nm)	0.382	0.428	0.331	0.358	0.412
Diffusion coefficient (Ds; 10^9^ m^2^/s)	1.030	0.706	1.957	1.333	0.718

**Table 4:** Optimum operating conditions and SF, MLR of commercial nanofiltration membranes.

Membrane	Dilution Factor	P (MPa)	Circulation Flow (L/h)	pH	SF	MLR
DL	40	1.2	500	7	0.074	0.088
DK	35	1.0	550	9	0.340	2.551
NF270	40	0.75	550	7	0.071	1.547
NF90	45	1.4	600	9	0.130	1.133

## Data Availability

Not applicable.
